# ESR Essentials: imaging of lymphoma—practice recommendations by the European Society of Oncologic Imaging

**DOI:** 10.1007/s00330-024-11213-5

**Published:** 2025-01-02

**Authors:** Doris Leithner, Emanuele Neri, Melvin D’Anastasi, Heinz-Peter Schlemmer, Michael Winkelmann, Wolfgang G. Kunz, Clemens C. Cyran, Dania Cioni, Evis Sala, Marius E. Mayerhoefer

**Affiliations:** 1 Department of Radiology, NYU Grossman School of Medicine, New York, USA; 2 Diagnostic and Interventional Radiology, Department of Translational Research, University of Pisa, Pisa, Italy; 3 Medical Imaging Department, Mater Dei Hospital, University of Malta, Msida, Malta; 4 Department of Radiology, German Cancer Research Center, Heidelberg, Germany; 5 Department of Radiology, University Hospital LMU Munich, Munich, Germany; 6 Department of Radiology, Universita Cattolica del Sacro Cuore, Rome, Italy; 7 Department of Radiology, Radiation Oncology and Hematology, Fondazione Policlinico Universitario A. Gemelli IRCCS, Rome, Italy; 8 Department of Biomedical Imaging and Image-guided Therapy, Medical University of Vienna, Vienna, Austria

**Keywords:** Lymphoma, Leukemia, Computed tomography, Positron emission tomography, Magnetic resonance imaging

## Abstract

**Abstract:**

Imaging is used for lymphoma detection, Ann Arbor/Lugano staging, and treatment response assessment. [^18^F]FDG PET/CT should be used for most lymphomas, including Hodgkin lymphoma, aggressive/high-grade Non-Hodgkin lymphomas (NHL) such as diffuse large B-cell lymphoma, and many indolent/low-grade NHLs such as follicular lymphoma. Apart from these routinely FDG-avid lymphomas, some indolent NHLs, such as marginal zone lymphoma, are variably FDG-avid; here, [^18^F]FDG PET/CT is an alternative to contrast-enhanced CT at baseline and may be used for treatment response assessment if the lymphoma was FDG-avid at baseline. Only small lymphocytic lymphoma/chronic lymphocytic leukemia (SLL/CLL) should exclusively undergo CT at baseline and follow-up unless transformation to high-grade lymphoma is suspected. While [^18^F]FDG PET/CT is sufficient to rule out bone marrow involvement in Hodgkin lymphoma, biopsy may be needed in other lymphomas. The 5-point (Deauville) score for [^18^F]FDG PET that uses the liver and blood pool uptake as references should be used to assess treatment response in all FDG-avid lymphomas; post-treatment FDG uptake ≤ liver uptake is considered complete response. In all other lymphomas, CT should be used to determine changes in lesion size; for complete response, resolution of all extranodal manifestations, and for lymph nodes, long-axis decrease to ≤ 1.5 cm are required.

**Key Points:**

- *[* ^*18*^ *F]FDG-PET/CT and contrast-enhanced CT are used to stage lymphoma depending on type.*
- *Imaging is required for staging, and biopsies may be required to rule out bone marrow involvement.*
- *For treatment response assessment, the 5-PS (Deauville) score should be used; in a few indolent types, CT is used to determine changes in lesion size.*

## Key recommendations

- Hodgkin lymphoma, all aggressive and many indolent Non-Hodgkin lymphomas (NHL) should undergo [^18^F]FDG-PET/CT for staging and response assessment; in small lymphocytic lymphoma/chronic lymphocytic leukemia (SLL/CLL) and non-FDG-avid indolent NHL, CT is used (Evidence level: high).
- Imaging is required for staging according to Ann Arbor or Lugano systems; biopsies may be required to rule out bone marrow involvement in all subtypes except Hodgkin lymphoma (Evidence level: high).
- For treatment response assessment, the 5-PS (Deauville) score that compares FDG uptakes of lymphoma manifestations and reference tissues on PET should be used; in SLL/CLL and non-FDG-avid indolent NHL, CT is used to determine changes in lesion size (Evidence level: high).

## Introduction

Lymphoma is a heterogeneous group of malignancies that arise from the lymphatic system. The 5th edition of the WHO classification of lymphoid neoplasms recognizes numerous distinct lymphoma subtypes [1], which differ in terms of symptoms, prognosis, and treatment options that now also include cutting-edge immunotherapies such as chimeric antigen receptor (CAR) T-cells for some subtypes [2].

**Fig. 1:**
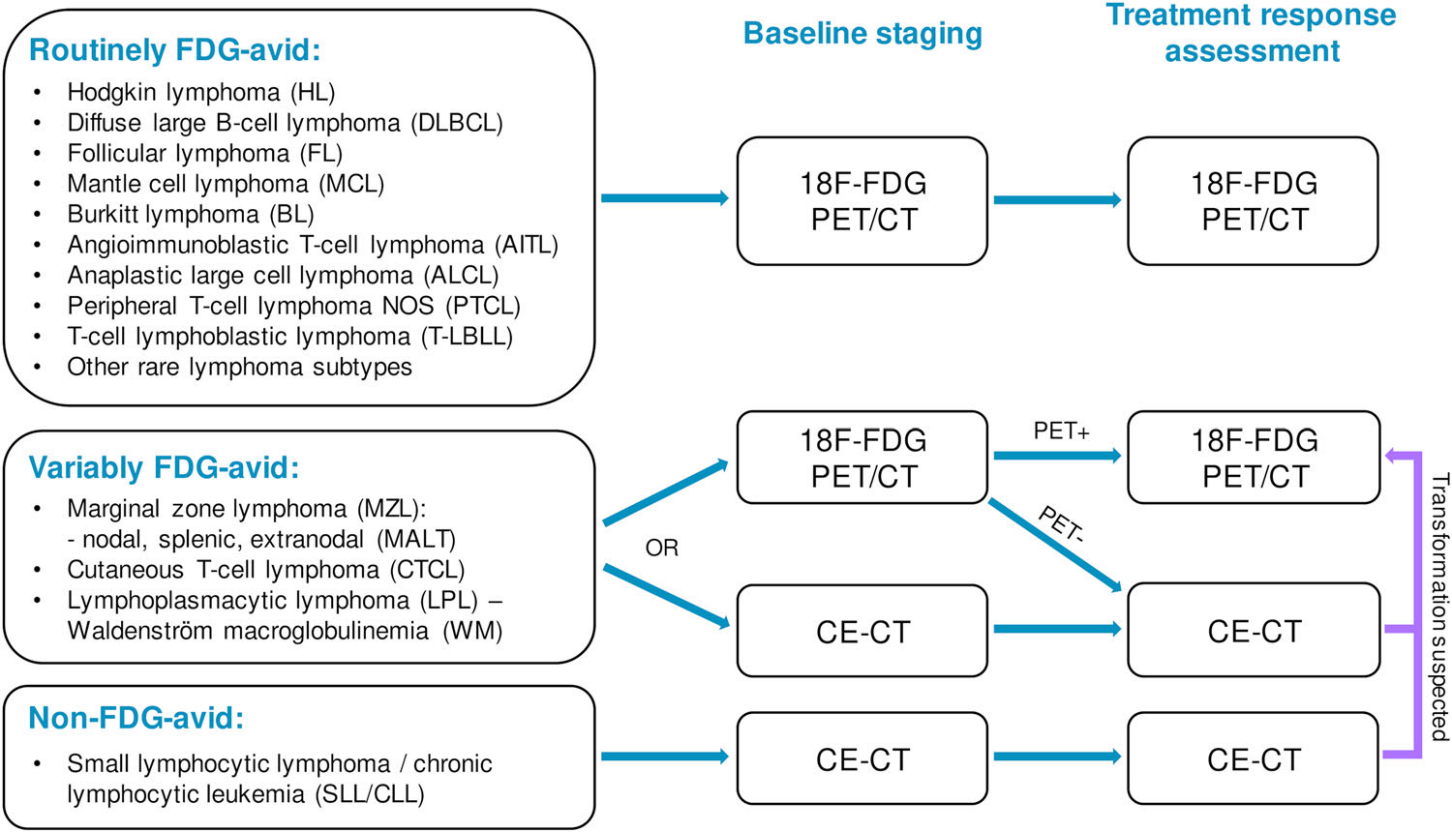
Imaging of lymphomas according to histopathological subtype

While detailed knowledge of the WHO classification is not required for clinical radiologists, familiarity with the most common lymphoma subtypes is essential for choosing the appropriate imaging test, and facilitates image interpretation. Beyond the classic division into Hodgkin (10%) and Non-Hodgkin lymphoma (NHL, 90%), the NHL categorization into fast-growing, aggressive, “high-grade” lymphomas such as diffuse large B-cell lymphoma (DLBCL, 30–58%), and slowly-growing, indolent, “low-grade” subtypes such as follicular lymphoma (20–25%) is of practical relevance (see Fig. 1).

Imaging plays a central role in the management of lymphoma; it is used for initial detection, staging, treatment response assessment, as well as for biopsy guidance [3, 4]. Two main imaging tests are currently used: contrast-enhanced (CE-)CT for assessment of lesion size/morphology, and PET/CT using 18F-fluorodeoxyglucose ([^18^F]FDG), which captures the lesions’ glucose metabolism.

In this article, we summarize current recommendations for stratification of lymphoma patients to [^18^F]FDG-PET/CT or CT, reporting/terminology, staging, and treatment response assessment.

### Imaging of lymphoma—state of the art

[^18^F]FDG-PET/CT is the preferred modality in the vast majority of patients because most lymphomas are hypermetabolic and because [^18^F]FDG-PET detects treatment response earlier than CT [3, 4]. According to the recent PRoLoG consensus [4], there are three lymphoma subtype categories in terms of [^18^F]FDG uptake (see Fig. 1):

- Routinely FDG-avid: Most lymphomas, including Hodgkin lymphoma, DLBCL, and follicular lymphoma as the most common subtypes. Here, PET/CT is used for staging and treatment response assessment.
- Non-FDG-avid: Small lymphocytic lymphoma (SLL)/chronic lymphocytic leukemia (CLL), for which CE-CT is the test of choice. PET/CT is only recommended if transformation to high-grade lymphoma (typically DLBCL; so-called Richter transformation) is suspected clinically. In such patients, a maximum standardized uptake value (SUVmax) > 5 is suspicious for transformation (negative and positive predictive values, 80% and 53%) [5], and biopsy is usually performed.
- Variably FDG-avid: Includes marginal zone lymphoma (MZL), cutaneous T-cell lymphomas, and (according to Lugano [3]) lymphoplasmacytic lymphoma/Waldenström macroglobulinemia. PET/CT may be performed at baseline, and if manifestations are FDG-avid, may also be used for response assessment. In non-FDG-avid cases, CE-CT is used to assess response, unless transformation is suspected.

Contrary to the above recommendations, patients may undergo CT rather than PET/CT for other reasons in routine clinical practice, for example, for initial assessment of lymphadenopathy prior to pathology workup, in patients with marked hyperglycemia, or in patients residing in remote areas without access to PET/CT. Whether diagnostic CE-CT or low-dose non-contrast CT should be used for PET/CT is still subject to debate, and regulations and institutional policies differ between and within countries. The PRoLoG consensus recommends that PET and CE-CT should be performed on the same scanner, but that for PET attenuation correction, a non-contrast CT series should be obtained [4].

MRI is currently the only standard of care for CNS lymphoma but may be used as a problem-solving tool in equivocal bone (marrow) lesions at treatment response assessment with PET/CT [3]. While not officially recommended in guidelines, whole-body MRI with diffusion-weighted sequences performs well in lymphoma [6–8], and is sometimes used in vulnerable populations where exposure to ionizing radiation is a concern, for example during pregnancy. Contrast-enhanced MRI is accepted in lieu of CE-CT for size measurements in many clinical trials but is not recommended for routine clinical practice.

PET/MRI, which entered the clinical stage a decade ago and showed non-inferiority or a slight advantage over PET/CT in lymphoma [9–11], is currently not mentioned in the Lugano classification, probably because it was published in 2014, and because PET/CT installations clearly outnumber PET/MRI installations. Still, in the authors’ experience, PET/MRI is usually accepted as an alternative to PET/CT, both in clinical routine and within clinical trials.

### Rules and pitfalls for image interpretation

**Fig. 2:**
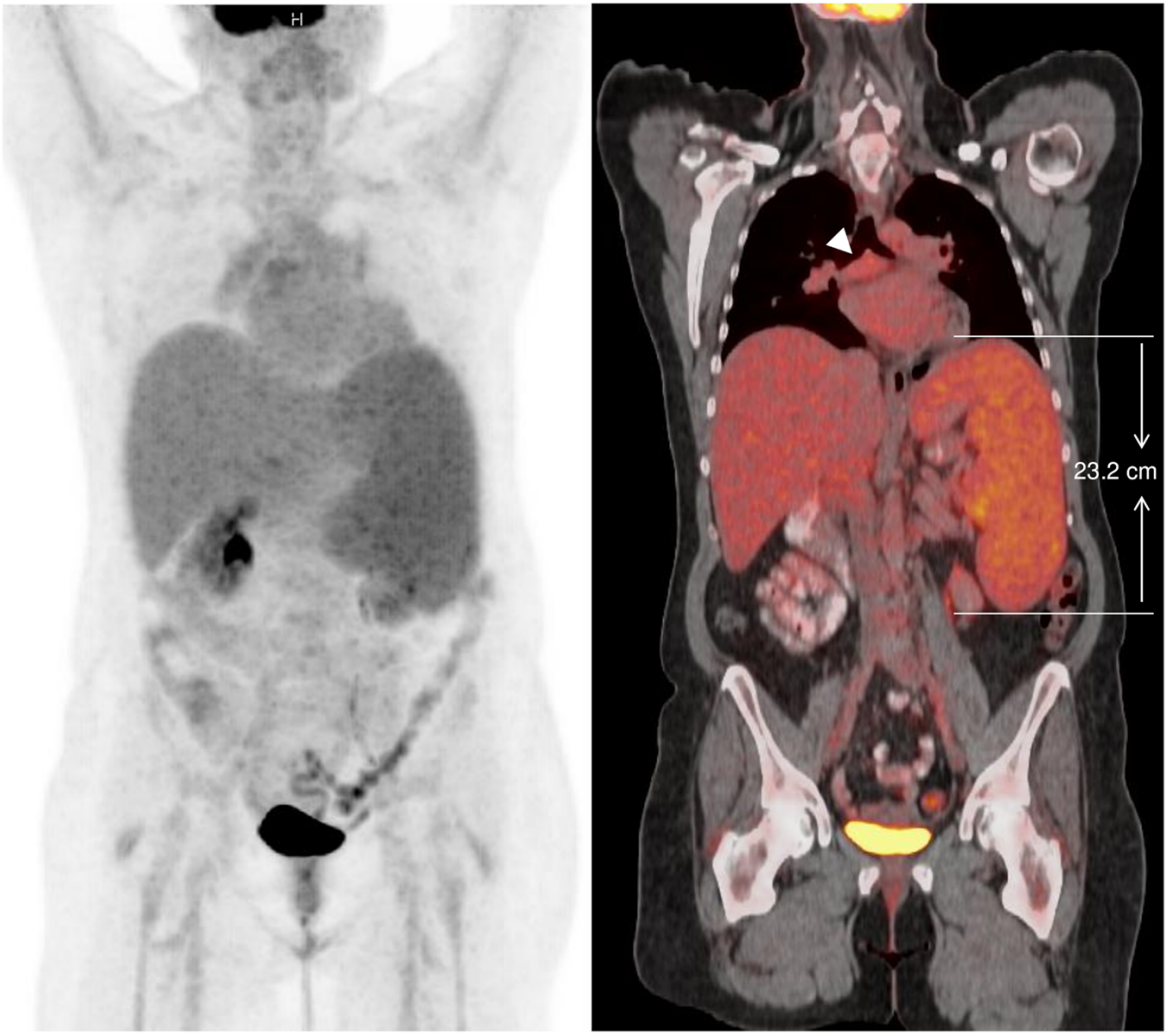
A 54-year-old female patient with splenic marginal zone lymphoma and baseline [^18^F]FDG-PET/CT. With 23.2 cm vertical diameter, the spleen is enlarged (cut-off: 13 cm), and splenic [^18^F]FDG uptake is also slightly higher on PET than the hepatic reference uptake; therefore, both metabolic and anatomic criteria for lymphoma involvement are met. There is also mildly hypermetabolic mediastinal lymphadenopathy (white arrowhead). The supra- and infradiaphragmatic nodal/lymphatic lymphoma involvement therefore suggests stage III disease. However, bone marrow involvement, which is not visible on PET/CT, was detected on iliac crest biopsy, and therefore, the patient has stage IV disease
- Bone and bone marrow: Lytic lesions and cortical destruction occur in a small percentage of cases, and are well-appreciated on CT and [^18^F]FDG-PET. Much more common, however, is the presence of focal or diffuse bone marrow involvement, which CT fails to detect in most cases. In Hodgkin lymphoma, bone marrow involvement is typically focal/multifocal, consistently FDG-avid, and sometimes, mildly sclerotic; therefore, PET/CT is sufficient to detect or rule out bone marrow involvement in Hodgkin lymphoma [3]. If focal FDG-avid bone marrow lesions are present in DLBCL, PET/CT is also sufficient for diagnosis. However, to rule out diffuse involvement in DLBCL, PET/CT is not sensitive enough, and therefore, bone marrow biopsy is required. While data suggest a high level of accuracy in follicular lymphoma [13], biopsy is currently recommended to rule out lymphoma in this and all other NHL subtypes [3].
- Gastrointestinal tract: Lymphoma may present as gastric/bowel wall thickening on CT, especially after oral water administration for distension. While in aggressive lymphomas, focal uptake on [^18^F]FDG-PET is consistently visible, lesion conspicuity may be reduced by physiologic, inflammatory, or drug-induced FDG uptake (e.g., after metformin intake). [^18^F]FDG-PET has poor sensitivity for indolent lymphomas such as gastric extranodal MZL (MALT) lymphoma [14], and CT is also severely limited when involvement primarily consists of superficial mucosal cell infiltrates. Mantle cell lymphoma frequently manifests as small intestinal polyps not seen on PET or CT. Endoscopy and biopsy are therefore the standard of care for GI tract evaluation in both gastric MALT lymphoma and mantle cell lymphoma [15, 16], and in other lymphoma subtypes when imaging is equivocal.
- Lungs: Lymphoma can show many different CT patterns, including solid and ground glass nodules, infiltrates, and consolidations. This can make the differentiation between lymphoma and infectious/inflammatory diseases, as well as between lymphoma and other pulmonary malignancies, difficult, especially since these entities may also be FDG-avid. Correlation with clinical and laboratory findings is therefore imperative, especially when it could affect staging.

Reporting of lymphoma differs from that of other cancers in various ways, both for morphological imaging with CT or MRI and for [^18^F]FDG PET. Key differences are:

- Terminology: Lymphoma usually presents as a systemic disease that, even at initial staging, frequently involves different anatomic sites, and lacks a primary tumor. Therefore, the terms “primary” and “metastasis” are discouraged, and the terms “nodal manifestations” when referring to lymphadenopathy (see below), or “extranodal manifestations” when referring to solid organ or soft tissue lesions, are used. When there is diffuse or multifocal organ disease, for example, in the bone marrow or spleen, “lymphoma involvement” is also appropriate.
- Anatomic regions: Sites of disease are broadly defined in lymphoma and include: right cervical, left cervical, right axillary, left axillary, right infraclavicular, left infraclavicular, mediastinal, hilar, periaortic, mesenteric, right pelvic, left pelvic, right inguinal/femoral, and left inguinal/femoral. The number of involved anatomic regions is important for staging, whereas the number of enlarged nodes within a region is irrelevant.
- Lymph nodes: Based on expert consensus [3], nodes with a long-axis diameter > 1.5 cm are suspicious for lymphoma (contrary to other cancers that use a short-axis diameter cut-off of 1.0 cm). Use of the term “bulk” depends on the subtype: ≥ 10 cm or > 1/3 of the transthoracic diameter in Hodgkin lymphoma, ≥ 6 cm for follicular lymphoma, and ≥ 6 or ≥ 10 cm in DLBCL [3], as measured on CT.
  For [^18^F]FDG-PET, any uptake above that of the surrounding tissue is regarded as FDG-avid and potentially suspicious for lymphoma; there is no SUVmax cut-off. In patients with biopsy-proven lymphoma, all FDG-avid nodes are by default suspicious for lymphoma; however, lesion location, size, morphology, treatment status, and change on serial imaging facilitate interpretation. Enlarged or non-enlarged peripheral nodes (i.e., cervical, axillary, and inguinal) are frequently mildly to moderately FDG-avid due to infection/inflammation, for example, in the neck during upper respiratory tract infections, and in the axillae secondary to vaccination [12]. While a fatty hilum on CT is not a reliable criterion for distinguishing between reactive/inflammatory nodes and lymphoma, it can be helpful for the assessment of mildly FDG-avid nodes. FDG-avid nodes without change on serial imaging are also more likely benign.
- Spleen: Lymphoma may present as focal manifestation(s) on CE-CT and PET or as diffuse disease. The criterion for lymphoma-associated splenomegaly is a vertical diameter > 13 cm on coronal CT, MRI, or PET (straight line from the level of the upper to the level of the lower pole). Measurements in the axial plane and oblique measurements are discouraged. The baseline [^18^F]FDG-PET criterion for splenic involvement is uptake > liver uptake; visual comparison is usually sufficient. As a lymphatic organ, the spleen is considered a nodal region for staging (see below, and Fig. 2).

### Staging of lymphoma

Staging is based on the Lugano or Ann-Arbor systems for the majority of lymphomas, which, in terms of imaging, are almost identical. The four stages of lymphoma are [3]:

- Stage I: Lymphadenopathy in a single anatomic region; or a single lymphoma manifestation in one extranodal site (stage IE).
- Stage II: Lymphadenopathy in > 1 anatomic region, but on the same side of the diaphragm; with or without limited contiguous manifestation in a single extranodal site (stage IIE).
- Stage III: Lymphadenopathy in anatomic regions on both sides of the diaphragm, or supradiaphragmatic lymphadenopathy with spleen involvement.
- Stage IV: Involvement of > 1 extranodal site, or diffuse or multifocal involvement of a single extranodal site (e.g., multiple liver or lung lesions, or diffuse bone marrow involvement). The presence and degree of concomitant lymphadenopathy is irrelevant.

Except for Hodgkin lymphoma and cases of obvious stage IV disease on imaging (e.g., multiple organ lesions), the limited accuracy of [^18^F]FDG-PET and CT for bone marrow (and for some subtypes, GI tract) involvement precludes definitive staging by imaging alone and requires correlation with pathology. Therefore, in the absence of pathology information, the use of Lugano/Ann Arbor stages in imaging reports is discouraged. Depending on institutional preferences, terminology such as “PET/CT-based” or “CT-based stage” may be acceptable. No Lugano/Ann Arbor stages are reported for SLL/CLL or cutaneous lymphomas, such as mycosis fungoides, which use other staging systems that are less dependent on imaging.

### Treatment response assessment: rules and pitfalls

[^18^F]FDG-PET/CT has revolutionized treatment response assessment in lymphoma and is used in all FDG-avid lymphomas at baseline. Clinical reports should provide SUVmax values of the most FDG-avid as well as the largest lymphoma manifestations and the semiquantitative 5-point Deauville score (5-PS) [3]. The 5-PS score compares the uptake in the most hypermetabolic/FDG-avid lymphoma manifestation on post-treatment PET to the respective uptake in the liver and blood pool (measured in the aortic arch or left atrium), as below:

- Score 1: No focal FDG uptake
- Score 2: FDG uptake ≤ blood pool uptake
- Score 3: FDG uptake > blood pool uptake, but ≤ hepatic uptake
- Score 4: FDG uptake > hepatic uptake, but ≤ 2x hepatic uptake
- Score 5: FDG uptake > 2x hepatic uptake, or new FDG-avid lymphoma manifestations

**Fig. 3:**
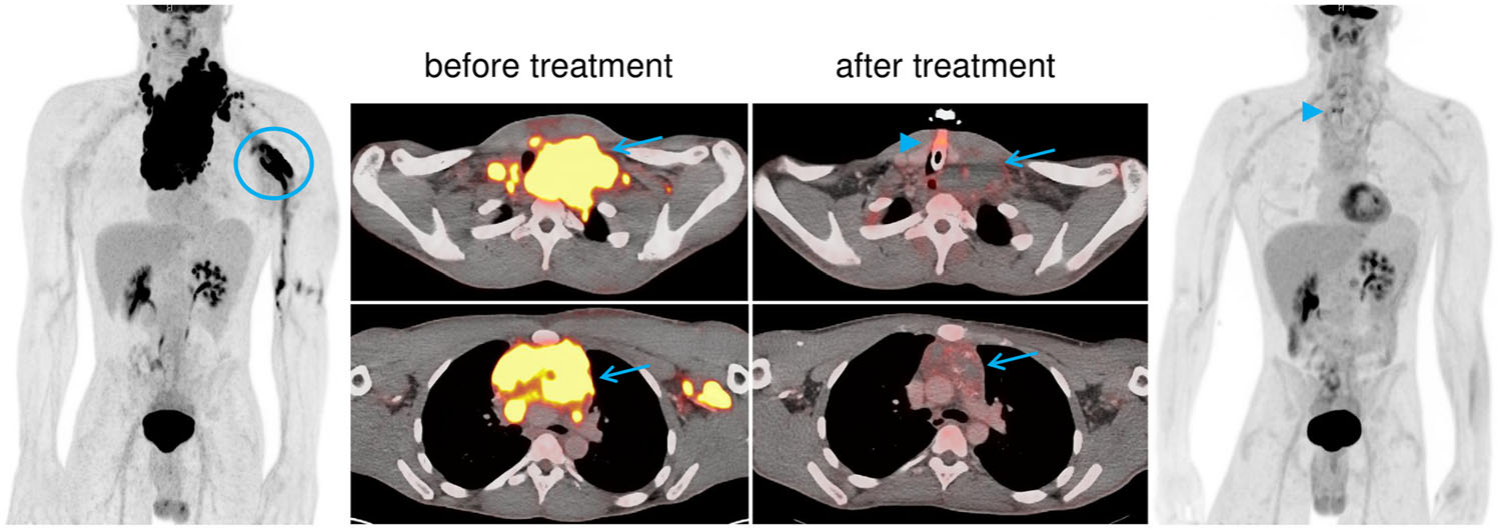
A 27-year-old male patient with diffuse large B-cell lymphoma. Before treatment (baseline), a markedly hypermetabolic nodal conglomerate extending from the anterior mediastinum to the neck is visible (blue arrows), and additional hypermetabolic neck nodes are also present (stage II). After treatment, the mass has markedly decreased, now includes necrotic components and calcifications, and shows residual uptake ≤ liver uptake, corresponding to a 5-PS (Deauville) score of 3 and consistent with complete (metabolic) response. The hypermetabolic mediastinal focus on PET after treatment (arrowheads) is related to a new tracheostoma. The marked, left axillary uptake on the maximum intensity projection before treatment is due to venous congestion and slow blood flow secondary to the mediastinal mass, with FDG accumulation in the axillary vessels (blue circle)

Based on the 5-PS, and the post-treatment time point, response categories are assigned (see Table 1, Fig. 3), which are used in both routine clinical practice and clinical trials. Generally, a 5-PS score ≤ 3 is regarded as complete metabolic response (CMR), whereas scores > 3 at end of treatment are consistent with viable residual lymphoma. A single 5-PS score per scan is required; readers may additionally provide separate scores for individual involved sites to facilitate report interpretation.

**Table 1:** Treatment response assessment criteria according to the Lugano classification, updated with PRoLoG consensus recommendations

	Metabolic criteria ([^18^F]FDG-PET/CT)	Anatomic criteria* (CE-CT and MRI)
Complete response (CR)	• 5-PS (Deauville) score 1–3, with or without residual lymphoma manifestation	• Resolution of extranodal manifestations and decrease of nodes to ≤ 1.5 cm long axis • Resolution of splenomegaly (to ≤ 13 cm vertical diameter)
Partial response (PR)	• 5-PS score 4–5 with decreased uptake compared to baseline, with residual manifestation(s) of any size Interim PET: responding disease End-of-treatment PET: residual lymphoma	• Residual lymphoma with overall ≥ 50% size decrease (based on sum of diameter products of six target lesions, SPD); and/or • Splenomegaly with ≥ 50% decrease beyond normal
Stable disease (SD)	• 5-PS score 4–5 with unchanged uptake compared to baseline, with residual manifestation(s) of any size	• Residual lymphoma manifestations with overall < 50% size decrease (SPD of six target lesions)
Progressive disease (PD)	• 5-PS score 4–5 with increased uptake intensity and/or metabolic tumor volume compared to baseline; or New hypermetabolic lymphoma manifestations	• Single node diameter product increase ≥ 50% (and at least +0.5 cm diameter) compared to nadir; or • New splenomegaly (at least +2 cm vertical diameter since baseline) • Increased splenomegaly: > 50% increase of enlargement beyond 13-cm cut-off (e.g., from 15 cm vertical diameter to > 16 cm)

* Intended chiefly for clinical trials

While the 5-PS score is based on visual comparison of post-treatment lesion uptake with reference tissue uptake, SUVmax measurements are now commonly used, especially if differences in uptake are subtle, and to distinguish between scores 4 and 5. Since the liver can sometimes show metabolic heterogeneity, the use of a large volume of interest is recommended [4]. Since SUVs may slightly differ depending on the PET scanner and acquisition protocol (e.g., injected activity and post-injection delay), it is desirable to perform serial scans using the same, or a similar, device and protocol, to ensure comparability.

Pitfalls for post-treatment [^18^F]FDG-PET/CT interpretation are mostly related to iatrogenic and non-iatrogenic infectious/inflammatory, as well as posttraumatic uptake, e.g., in pneumonia, colitis, arthritis, reactive/inflammatory nodes, fractures and hematoma, vaccination, biopsies and surgeries, catheters and tubes, and thrombi (see Fig. 3). FDG-avid thymic rebound may also be challenging in patients with mediastinal lymphoma; diffusion-weighted and chemical shift MRI may be helpful for differentiation. Finally, treatment-related diffuse splenic hypermetabolism and granulocyte colony-stimulating factor (G-CSF)-induced diffuse bone marrow hypermetabolism are commonly observed, and must not be mistaken for disease progression.

On CT, complete response requires resolution of all extranodal manifestations and regression to normal size for both lymph nodes (to ≤ 1.5 cm long-axis diameter) and spleen (to ≤ 13 cm vertical diameter) in both clinical practice and clinical trials. A more granular assessment for distinguishing between stable disease and partial response and stable disease and progression requires the definition of target lesions, which is the standard for patients enrolled in clinical trials, but rarely done outside of trials. Therefore, unless response is obvious, e.g., in case of marked tumor burden decrease with few residual lesions or marked tumor burden progression with/without new manifestations, response classification terminology should be used with caution in clinical reports.

### Current trends and developments

While several studies have confirmed the value of the 5-PS (Deauville) score for outcome prediction [17, 18], an increasing number of studies suggest that true quantitative PET metrics, in particular SUVmax and metabolic tumor volume (MTV), may be superior, both in patients treated with standard (immuno-)chemotherapy [19, 20] and in patients receiving novel treatments such as CAR T-cells [21, 22]. Notably, in DLBCL, a SUVmax decrease ≤ 66% after two immunochemotherapy cycles suggested poor prognosis in a large clinical trial [23]. For automated lesion detection/segmentation, extraction of quantitative PET metrics, and distinguishing between CMR and non-CMR, deep-learning algorithms are currently being evaluated [24, 25].

Since [^18^F]FDG-PET is limited in some indolent lymphomas, and not recommended for CNS lymphoma, other PET tracers have been evaluated. Among them, the CXCR-targeting tracer 68Ga-Pentixafor has shown promise, especially in marginal zone, mantle cell, and lymphoplasmacytic lymphoma, SLL/CLL, and (due to a lack of physiologic uptake in the brain) also CNS lymphoma [26–30].

### Summary statement

Lymphoma is a large and heterogeneous group of lymphoid neoplasms that differ in terms of clinical presentation, prognosis, and treatment options. [^18^F]FDG-PET/CT is the recommended baseline imaging test for all lymphomas except the indolent SLL/CLL, which is evaluated with CE-CT unless transformation to aggressive lymphoma is suspected. Staging (Lugano/Ann Arbor) is based chiefly on imaging in the vast majority of lymphomas. In limited disease (stages I-II), lymphoma manifestations are only present on one side of the diaphragm, whereas in advanced disease, lymphoma manifestations are either found on both sides of the diaphragm (stage III), or there is multifocal or diffuse involvement of one or more extranodal organs/tissues (stage IV). In all lymphomas that are FDG-avid at baseline, PET/CT should also be used for treatment response assessment. PET response is based on the 5-PS (Deauville) score, which uses the hepatic FDG uptake as reference to distinguish between treated disease (scores 1–3, FDG uptake ≤ liver) and residual viable lymphoma (scores 4–5, FDG uptake > liver) at end of treatment. Quantitative [^18^F]FDG-PET metrics such as SUVs and MTV may possibly replace the 5-PS score in future guidelines and may be included in predictive and prognostic models.

### Patient summary

Lymphoma is a type of blood cancer that can either be fast-growing (aggressive) or slowly growing (indolent). While lymph nodes are most commonly affected, lymphoma can involve many different body parts. Imaging tests such as positron emission tomography (PET) and computed tomography (CT) are essential to determine the extent of lymphoma and its spread throughout the body. Sometimes, additional bone marrow biopsy may be needed. PET and CT can also determine whether treatment has been successful. PET can inform earlier than CT if there is still viable lymphoma at the end of treatment.

## Abbreviations

CAR: Chimeric antigen receptor
CLL: Chronic lymphocytic leukemia
DLBCL: Diffuse large B-cell lymphoma
MTV: Metabolic tumor volume
NHL: Non-Hodgkin lymphomas
SLL: Small lymphocytic lymphoma
SUVmax: Maximum standardized uptake value
